# H_2_O_2_ treatment or serum deprivation induces autophagy and apoptosis in naked mole-rat skin fibroblasts by inhibiting the PI3K/Akt signaling pathway

**DOI:** 10.18632/oncotarget.13321

**Published:** 2016-11-12

**Authors:** Shanmin Zhao, Li Li, Shiyong Wang, Chenlin Yu, Bang Xiao, Lifang Lin, Wei Cong, Jishuai Cheng, Wenjing Yang, Wei Sun, Shufang Cui

**Affiliations:** ^1^ Laboratory Animal Centre, Second Military Medical University, Shanghai, China; ^2^ Department of Training, Second Military Medical University, Shanghai, China; ^3^ Informatization Office, Second Military Medical University, Shanghai, China

**Keywords:** naked mole-rats, autophagy, apoptosis

## Abstract

Naked mole-rats (NMR; *Heterocephalus glaber*) display extreme longevity and resistance to cancer. Here, we examined whether autophagy contributes to the longevity of NMRs by assessing the effects of the PI3K/Akt pathway inhibitor LY294002 and the autophagy inhibitor chloroquine (CQ) on autophagy and apoptosis in NMR skin fibroblasts. Serum starvation, H2O2 treatment, and LY294002 treatment all increased the LC3-II/LC3-I ratio and numbers of double-membraned autophagosomes and autophagic vacuoles, and decreased levels of p70S6K, p-Akt^Ser473^, and p-Akt^Thr308^. By contrast, CQ treatment decreased p70S6K, Akt^Ser473^, and Akt^Thr308^ levels. The Bax/Bcl-2 ratio increased after 12 h of exposure to LY294002 or CQ. These data show that inhibiting the Akt pathway promotes autophagy and apoptosis in NMR skin fibroblasts. Furthermore, LY294002 or CQ treatment decreased caspase-3, p53, and HIF1-α levels, suggesting that serum starvation or H2O2 treatment increase autophagy and apoptosis in NMR skin fibroblasts by inhibiting the PI3K/Akt pathway. CQ-induced inhibition of late autophagy stages also prevented Akt activation and induced apoptosis. Finally, the HIF-1α and p53 pathways were involved in serum starvation- or H2O2-induced autophagy in NMR skin fibroblasts.

## INTRODUCTION

Naked mole-rats (NMR; *Heterocephalus glaber*) are long-lived rodents with a maximum lifespan of 30 years, which is about 5 times longer than similarly-sized mice [1]. They also experience negligible declines in bodily functions as they age, suggesting that anti-aging processes may occur in NMRs [2]. Interestingly, NMRs show a profound resistance to cancer, the rates of which vary widely amongst mammals [3–5]. NMRs can tolerate hypoxic conditions with 3% O_2_, and brain slices from NMRs display extreme tolerance to hypoxia compared to slices from other mammals [4, 6] due to the evolution of unique neuroprotective mechanisms [7]. Similar to other long-lived species, fibroblasts from NMRs are extremely tolerant to a broad spectrum of cytotoxins, including heat, heavy metals, DNA-damaging agents, and xenobiotics [8–10]. These unusual rodents provide novel insights into the mechanisms involved in aging [11]. Studies by Seluanov and colleagues have identified specific mechanisms that contribute to cancer resistance in NMRs. For example, NMR fibroblasts are hypersensitive to contact inhibition, a phenomenon termed “early contact inhibition” [4]. That group has also shown that NMRs have evolved a higher concentration of high-molecular-mass hyaluronan in the skin to provide the skin elasticity needed for life in underground tunnels, which may confer added benefits of cancer resistance and longevity [12]. Thus, NMRs have evolved physiological and biochemical processes that dramatically improve their health and extend their lifespans. Understanding the cellular and molecular mechanisms underlying these effects may provide insights into human disease.

Autophagy, the cellular process that mediates lysosomal degradation of long-lived cytoplasmic proteins, is initiated during periods of differentiation, starvation, or stress, including oxidative stress, endoplasmic reticulum stress, and the accumulation of protein aggregates [13– 16]. Autophagy, which is widely regarded as one of the most effective processes for slowing down the ageing process [17], plays important and complex roles in cancer resistance, resistance to ageing, and hypoxia [18]. We previously reported that autophagy is upregulated in NMRs compared to short-lived mice. In addition, Judy *et al.* showed that NMRs maintain high levels of autophagy for most of their lifespan [19]. These studies indicate that autophagy may contribute significantly to long lifespans in NMRs. Nutrient starvation and H_2_O_2_ induced high levels of autophagy in NMR skin fibroblasts and hepatic stellate cells (HSCs) from NMRs [20, 21]. Rodriguez *et al.* [22] also showed that, under conditions of serum deprivation, LC3-II/LC3-I ratios, which are indicative of vacuole development, were approximately two-fold higher in NMR cells than in cells from shorter-lived mice. Skin fibroblasts serve as a model for studying the chronological and biological aging of organisms according to polygenic predisposition and environmental etiopathology [23] due to their involvement in proliferation and migration in response to chemotactic, mitogenic, and modulatory cytokines [24]. Skin fibroblasts also have many applications in aging research, tissue engineering, cell nuclear transfer, and cell reprogramming. In this study, we treated NMR skin fibroblasts with LY294002 and chloroquine (CQ), which specifically inhibit PI3K and autophagy, respectively, and the examined the expression of genes involved in the PI3K/Akt signaling pathway and apoptosis. We found that inhibition of the PI3K/Akt signaling pathway increased autophagy in NMR skin fibroblasts.

## RESULTS

### Serum starvation or H_2_O_2_ treatment induce autophagy and apoptosis in NMR skin fibroblasts

**Figure 1:**
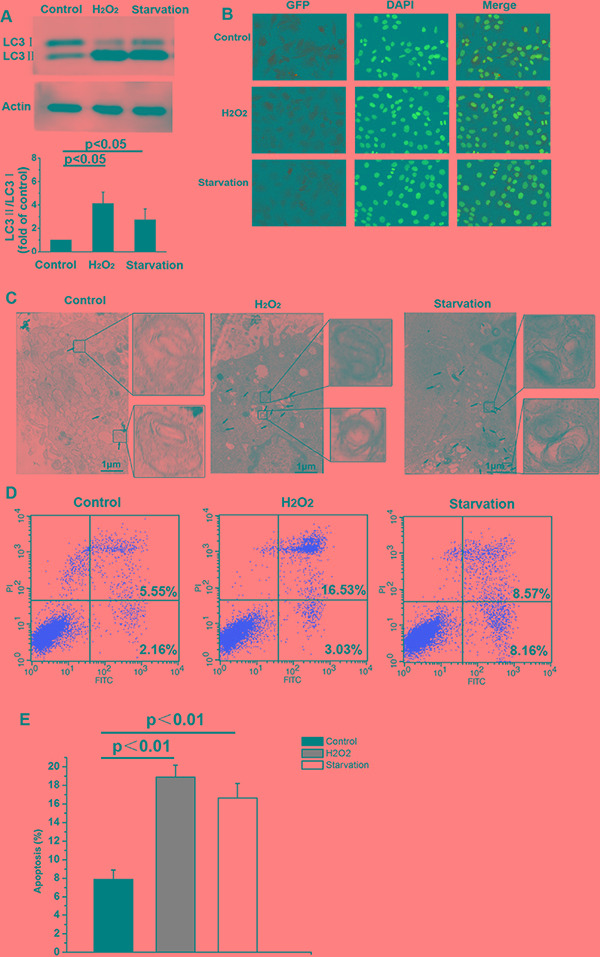
Serum starvation or H_2_O_2_ treatment induce autophagy and apoptosis in NMR skin fibroblasts (**A**) LC3-I and LC3-II levels were detected by Western blot in the untreated control and experimental groups after 12 h of treatment. β-actin served as a loading control. (**B**) Immunofluorescent labeling of LC3 in NMR skin fibroblasts following 12 h of serum starvation or H_2_O_2_ treatment. Coronal sections are labeled with an anti-LC3 antibody (green) and DAPI (blue; all panels, 200 × magnification). (**C**) Representative electron micrograph images showing autophagic vacuoles in each group. Arrows indicate autophagosomes. (**D**) Flow cytometric analysis of Annexin V-FITC and PI stained cells. Annexin V-positive, PI-negative cells are considered early apoptotic cells. Annexin V-positive, PI-positive cells are considered late apoptotic cells. (**E**) Bar graph showing early and late apoptotic cell percentages. Values represent means ± standard error.

As shown in Figure 1A, 12 h of H_2_O_2_ treatment or serum starvation increased the LC3-II/LC3-I ratio in skin fibroblasts compared to untreated controls. LC3-II levels and the LC3-II to LC3-I ratio correlate with autophagosome numbers in mammalian cells [25– 27]. Immunohistochemistry revealed that numbers of autophagic vacuoles increased in cells subjected to 12 h of serum starvation or H_2_O_2_ treatment as compared to control cells (Figure 1B). Furthermore, electron microscopy revealed a higher number of autophagosomes with double membranes in serum starved or H_2_O_2_ treated cells than in control cells (Figure 1C). These data indicate that serum starvation or H_2_O_2_ treatment induces autophagy. Serum starvation or H_2_O_2_ treatment also increased apoptosis compared to the control group (Figure 1D and E, *p* < 0.05).

### Inhibition of the PI3K/Akt signaling pathway increases serum starvation or H_2_O_2_-induced autophagy in skin fibroblasts

**Figure 2:**
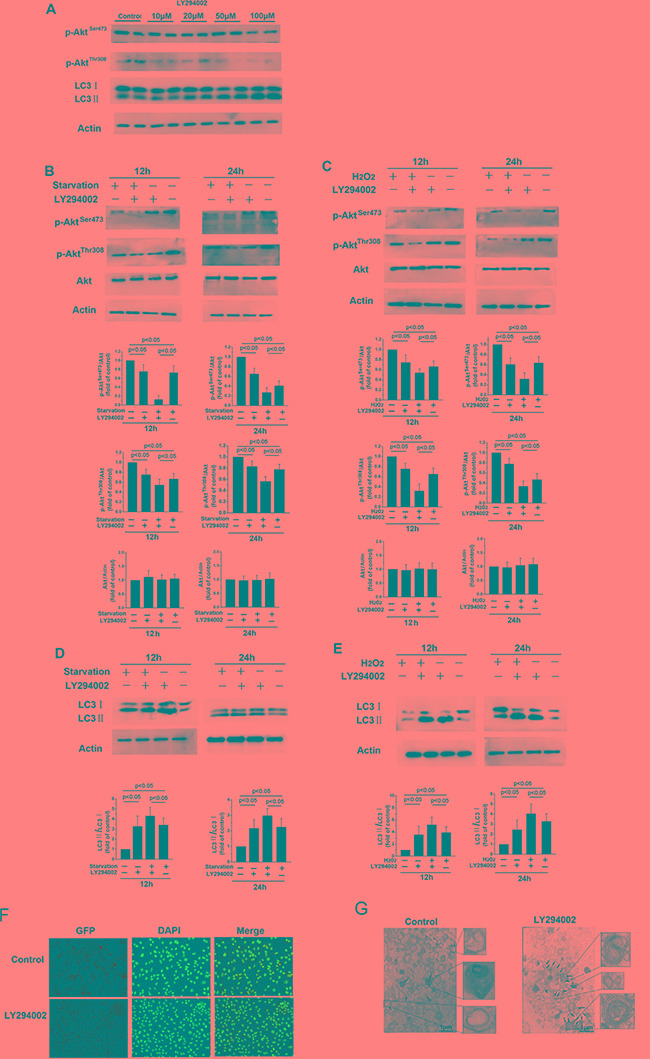
LY294002-induced inhibition of the PI3K/Akt signaling pathway increases autophagy in skin fibroblasts (**A**) Western blot showing protein levels in skin fibroblasts after 12 h of treatment with different concentrations of LY294002 for 12 h. (**B, C, D,** and **E**) Treatment with 20 μM LY294002 decreased p-Akt^Ser473^ and p-Akt^Thr308^ levels relative to total Akt levels, and increased LC3-II levels and the LC3-II/LC3-I ratio, after 12 or 24 h of serum starvation or H_2_O_2_ treatment. Bar graphs represents mean relative expression of protein normalized to untreated controls. (**F**) Immunofluorescence images of LC3 in skin fibroblasts treated with or without 20 μM LY294002 for 12 h. The coronal sections are labeled with an anti-LC3 antibody (green) and DAPI (blue; all panels, 100× magnification). (**G**) Representative electron micrograph images showing autophagic vacuoles in cells treated with or without 20 μM LY294002 for 12 hours. Arrows indicate autophagosomes.

The phosphorylation status of Akt, which is indicative of PI3K activity, was examined by Western blot. Treatment with LY294002, a PI3K inhibitor, decreased phosphorylated Akt levels and dose-dependently increased LC3-II levels (Figure 2A). To further investigate the role of the PI3K/Akt pathway in autophagic activation, we examined the effects of LY294002 on starvation- and H_2_O_2_-induced autophagy. p-Akt^Ser473^ and p-Akt^Thr308^ levels decreased relative to total Akt levels in fibroblasts when autophagy was activated by serum starvation or H_2_O_2_ treatment (Figure 2B and 2C, *p* < 0.05). More importantly, p-Akt^Ser473^ and p-Akt^Thr308^ levels decreased further in fibroblasts treated with 20 μM LY294002, while the LC3-II/LC3-I ratio increased (Figure 2D and 2E, *p* < 0.05). Immunohistochemistry revealed that numbers of autophagic vacuoles in cells increased 24 h after exposure to LY294002 compared to control cells (Figure 2F). In addition, the number of double-membraned autophagosomes increased in the LY294002 treatment group compared to the control group (Figure 2G). Together, these data indicate that inhibition of the PI3K/Akt pathway activates autophagy.

### Blocking late autophagy stages prevents Akt activation

**Figure 3:**
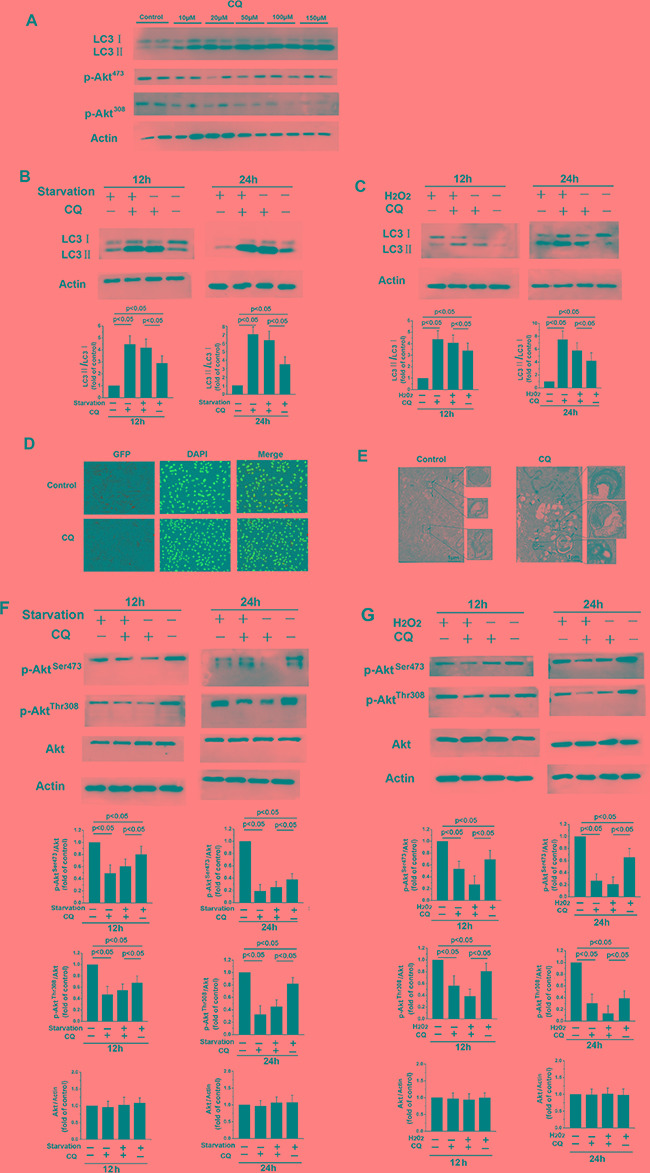
CQ-induced inhibition of late-stage autophagy prevents Akt activation (**A**) Western blot showing expression levels of LC3, Akt, p-Akt^Ser473^, and p-Akt^Thr308^ levels after 12 h of treatment with the indicated concentrations of CQ. β-actin served as a loading control. (**B, C, F,** and **G**) 12 or 24 h of treatment with 20 μM CQ decreased p-Akt^Ser473^ and p-Akt^Thr308^ protein levels relative to total Akt and increased LC3-II levels and the LC3-II/LC3I ratio. Bar graphs show mean relative protein levels normalized to β-actin/Akt. (**D**) Immunofluorescence images of LC3 in skin fibroblasts following 12 h of treatment with or without 20 μM CQ. The coronal sections are labeled with an anti-LC3 antibody (green) and DAPI (blue; all panels, 100× magnification). (**E**) Representative electron micrograph images showing autophagic vacuoles in cells treated with or without 20 μM CQ for 12 h. Arrows indicate autophagosomes.

Chloroquine (CQ) is an inhibitor of autophagic flux that prevents autophagosome-lysosome fusion and lysosomal protein degradation by raising the lysosomal pH in the later phases of the process [28]. As shown in Figure 3A, Western blot revealed that treatment with various concentrations of CQ increased LC3-II levels. Furthermore, treatment with 20 μM CQ increased the LC3-II/LC3-I ratio in fibroblasts after starvation or H_2_O_2_ treatment (Figure 3B and 3C). Numbers of autophagic vacuoles (Figure 3D) and double-membraned autophagosomes (Figure 3E) also increased after CQ treatment, indicating that CQ suppresses the fusion of autophagosomes and lysosomes. p-Akt^Ser473^ and p-Akt^Thr308^ levels also decreased when starvation- or H_2_O_2_-induced autophagy was inhibited by CQ, while total Akt levels were unchanged (Figure 3F and 3G). These results indicate that blocking autophagy with CQ decreases PI3K/Akt signaling pathway activity in NMR skin fibroblasts.

### LY294002- or CQ-induced inhibition of PI3K/Akt signaling promotes apoptosis in skin fibroblasts

**Figure 4:**
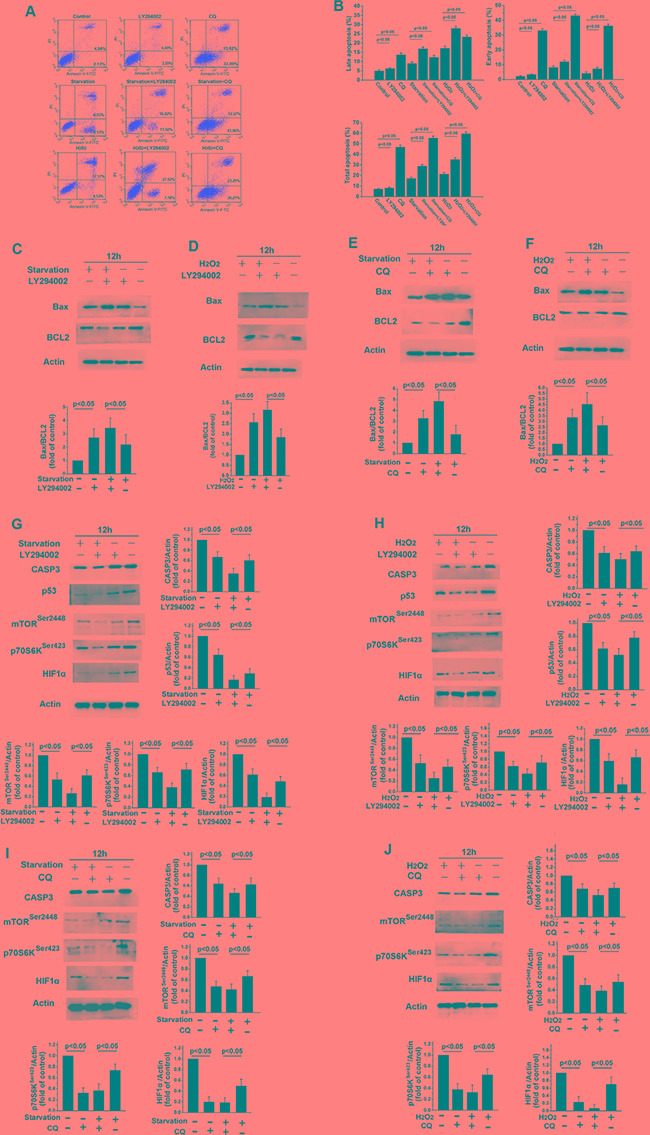
LY294002-induced inhibition of PI3K/Akt signaling increases apoptosis in skin fibroblasts (**A**) Flow cytometric analysis revealed that 12 h of treatment with LY294002 or CQ increased early and late apoptosis rates, and further increased starvation- or H_2_O_2_ treatment-induced increases in apoptosis rates, in skin fibroblasts. (**B**) Bar graph showing early and late apoptotic cell percentages. Means ± standard error are shown. (**C–J**) Western blots revealed that Bax levels increased, while Bcl2, p70S6K, p53, HIF1-α, and caspase-3 levels decreased, following 12 h of serum starvation or H_2_O_2_ treatment with or without LY294002 or CQ. Bar graphs show mean relative protein levels normalized to β-actin.

To assess the role of PI3K/Akt signaling in apoptosis, apoptosis and Bcl-2 and Bax levels were examined following treatment with the PI3K pathway inhibitor LY294002. As shown in Figure 4A, 12 h of LY294002 treatment clearly increased apoptosis in skin fibroblasts (Figure 4B, *p* < 0.05). LY294002 also increased early and late apoptosis rates in fibroblasts after H_2_O_2_ treatment or starvation (Figure 4B, *p* < 0.05). Bax levels increased, while Bcl-2 levels decreased, in fibroblasts treated with LY294002 (Figure 4C and 4D, *p* < 0.05). The balance between the expression of the pro-apoptotic protein Bax and the anti-apoptotic protein Bcl-2 is a critical in triggering cellular apoptosis, and changes in the levels of these proteins are indicative of apoptosis rates [29]. We also found that LY294002 treatment reduced the activity of caspase-3, another key regulator of apoptosis (Figure 4G and 4H, *p* < 0.05). These data show that inhibition of the survival kinase Akt can trigger apoptosis. Levels of p53 and HIF1-α also decreased when skin fibroblasts were treated with LY294002 for 12 h (Figure 4G and 4H, *p* < 0.05). In addition, LY294002 treatment decreased p-mTOR^Ser2448^ and p70S6K^ser423^ levels compared to untreated fibroblasts (Figure 4G and 4H, *p* < 0.05). As shown in Figure 4A, CQ-induced inhibition of autophagy and PI3K/Akt signaling also increased apoptosis in skin fibroblast. CQ treatment also increased Bax levels, decreased Bcl2 levels (Figure 4E and 4F, *p* < 0.05), and increased early and late apoptosis as measured by flow cytometry (Figure 4B, *p* < 0.05). These data revealed that exposure of cells to CQ increased H_2_O_2_- or starvation-induced early and late apoptosis rates. Levels of p53, HIF1-α, p-mTOR^Ser2448^, p70S6K^ser423^, and caspase-3 were also reduced in skin fibroblasts treated with CQ for 12 h (Figure 4I, 4J).

## DISCUSSION

Autophagy is essential for cancer cell survival under conditions of nutrient starvation, hypoxia, or chemotherapeutic stress [30]. In this study, we demonstrated that serum starvation and H_2_O_2_ treatment induced autophagy in NMR skin fibroblasts, which is in agreement with previous studies suggesting that autophagy may be linked to longevity [31]. Apoptosis rates increased 12 h after H_2_O_2_ treatment or serum starvation compared to controls, and the Bax/Bcl-2 ratio increased in skin fibroblasts after autophagy was inhibited by CQ. A recent study reported that pretreatment with the class III PI3K inhibitor 3-Methyladenine (3-MA) or knockdown of the autophagy-related gene Beclin-1 also promoted paclitaxel-induced apoptosis in A549 cells [32]. These data indicate that autophagy may protect against nutrient starvation- and H_2_O_2_ treatment-induced apoptosis in NMR cells.

We found that LY294002-induced inhibition of the PI3K/Akt pathway, as evidenced by the dephosphorylation of Akt ^Ser473^ and Akt^Thr308^, increased autophagy in skin fibroblasts, indicating that the PI3K/Akt signaling pathway is involved in serum starvation- or H_2_O_2_ treatment-induced autophagy in NMR skin fibroblasts. The PI3K/Akt/mTOR signaling pathway also inhibits autophagy in many types of human cancer cells, including ovarian cancer [33], HepG2 [34], prostate cancer [35], gastric cancer [36], and colorectal cancer cells [37]. However, other reports found that the PI3K/Akt pathway promotes autophagy in acute promyelocytic leukemia NB4 cells [38] and cervical carcinoma HeLa cells [39]. This suggests that carcinogenesis may alter the effects of PI3K/Akt pathway activity on autophagy. Akt is overexpressed in various cancers [40, 41], and the upregulation of phosphorylated Akt promotes cancer cell survival and angiogenesis [42]. For example, phosphorylated Akt levels are increased in human breast adenoma MDA-MB-231 cells [43] and in three HCC cell lines (HepG2, Hep3B and Huh7) [44] under starvation conditions. In addition, H_2_O_2_ treatment substantially increases total and phosphorylated Akt protein levels in human colorectal cancer SNU-407 cells [45], neuroblastoma SH-SY5Y cells [46], and human squamous cell carcinoma A431 cells [47]. In this study, we found that Akt phosphorylation at Ser473 and Thr308 decreased after nutritional starvation or H_2_O_2_ treatment in NMR skin fibroblasts. The same effect has been observed in other non-tumor cells, including 7702 human normal liver cells [44], mouse primary hippocampal neurons [48], and rat primary Leydig cells [49]. Thus, the same environmental stimulus, be it H_2_O_2_ treatment or serum starvation, has different effects on Akt expression in cancer cells than in normal cells. Akt signaling pathway activity is important for cancer cell proliferation and survival. In response to H_2_O_2_ or starvation, autophagy is increased in normal cells via downregulation of Akt signaling pathway activity, which normally inhibits autophagy. However, the Akt signaling pathway is upregulated in response to such environmental stresses in cancer cells, ultimately decreasing apoptosis and increasing cell cycle progression to promote cell survival.

The PI3K/Akt signaling pathway is a classical pro-survival and anti-apoptosis pathway [50]. The Bax/Bcl-2 ratio, which is indicative of apoptotic activity, increased after serum starvation or H_2_O_2_ treatment in skin fibroblasts. Additionally, apoptosis and Bax/Bcl-2 ratios increased after skin fibroblasts were treated with LY294002, which inhibits the PI3K/Akt pathway. CQ-induced inhibition of autophagy not only markedly suppressed PI3K/Akt pathway activity, but also increased apoptosis. Therefore, inhibition of the PI3K/Akt pathway might increase H_2_O_2_- or serum starvation-induced apoptosis. These data suggest that CQ exerts its effects on autophagy and apoptosis in fibroblasts by inhibiting the PI3K/Akt signaling pathway. In addition, inhibition of PI3K/Akt signaling dramatically decreased anti-apoptotic Bcl-2 and caspase-3 protein levels and increased pro-apoptotic Bax levels. These data suggest that inhibition of PI3K/Akt signaling induces apoptosis by regulating anti-apoptotic and pro-apoptotic proteins. Furthermore, inhibition of Akt decreased mTOR and p70S6K phosphorylation levels, indicating that the Akt/mTOR/p70S6K signaling pathway may be at least partially responsible for stress-induced apoptosis.

Multiple distinct pro-autophagic stimuli, including nutrient deprivation, induce Mdm2-dependent proteasomal degradation of p53 [51]. Recently, p53 has also emerged as a critical regulator of cell metabolism and autophagy, both of which are critical for cell proliferation and survival [52]. We previously found that serum starvation or H_2_O_2_ treatment decrease p53 expression in skin fibroblasts. In addition, PI3K/Akt pathway activity promotes ubiquitination and degradation of p53 [53]. In this study, p53 expression decreased when PI3K/Akt signaling was inhibited, indicating that H_2_O_2_ or serum starvation promotes p53 degradation via the PI3K/Akt pathway, and that inhibition of Akt may promote p53 degradation. Because inhibition of PI3K/Akt signaling and decreased p53 expression increased autophagy, it is possible that starvation or H_2_O_2_ treatment induce autophagic cell death in a p53-independent manner in NMR skin fibroblasts.

NMR skin fibroblasts are adapted to hypoxic environments and are often cultured under controlled hypoxia conditions (3% O_2_) [4, 12]. The best-characterized response to hypoxia is the induction of hypoxia-inducible factor (HIF)-1 [54]. HIF-1, a transcription factor, is the dominant regulator of hypoxia-mediated radioresistance [55, 56]. In addition to oxygen-dependent regulation, HIF-1 is regulated through oxygen-independent PI3K/Akt/mTOR pathway mechanisms [56–58]. Akt activation increases HIF-1α expression by increasing its translation under both normoxic and hypoxic conditions [59]. In this study, HIF1-α expression decreased in parallel with p-Akt levels in skin fibroblasts treated with LY294002 or CQ, suggesting that HIF-1α expression may be regulated by Akt-dependent mechanisms.

In conclusion, we demonstrated in the present study that serum starvation and H_2_O_2_ treatment induce autophagy and apoptosis in NMR skin fibroblasts, and that the PI3K/Akt/mTOR signaling pathway inhibits autophagy in these cells. Furthermore, our results suggest that the HIF-1α and p53 pathways are involved in the induction of autophagy in skin fibroblasts.

## MATERIALS AND METHODS

### Cell culture and reagents

Primary NMR skin fibroblasts were isolated from 3 neonatal naked mole-rats on postnatal day 1 using methods similar to those reported previously [4]. Naked mole-rats were obtained from the Department of Zoology at the University of Cape Town and maintained at the laboratory animal center of the Second Military Medical University. The handling of animals and study procedures were in accordance with the current Chinese regulation “GB14925-2010 Laboratory animal requirements of environment and housing facilities” (Chinese version). The complete protocol was reviewed and approved by the Institutional Animal Care and Use Committee of the Second Military Medical University. NMR cells were maintained at 35°C in Dulbecco's Modified Eagle's Medium-low glucose (DMEM; GIBCO, USA) supplemented with 10% fetal bovine serum (FBS; GIBCO, USA) and 100 units/mL penicillin/streptomycin in a humidified incubator under 92% N_2_, 5% CO_2_, and 3% O_2_. All cell lines were used at early passages (7–12 population doublings). To investigate the effects of serum starvation, cells were incubated in the absence of glucose (DMEM-no glucose, GIBCO, Invitrogen) and serum. Cells were then treated with or without the PI3K/Akt inhibitor LY294002 (20 μM) for 12 h and 24 h, respectively, and harvested for examination by electron microscopy, cell apoptosis assay, or Western blotting analysis. The same approach was used to treat cells with or without the apoptosis inhibitor CQ (20 μM).

LY294002 (PI3K/Akt inhibitor) was purchased from Cyagen Biosciences (Guangzhou, China). CQ powder was purchased from Sigma (St. Louis, MO, USA). The antibodies against Akt, p-Akt (p-Ser473), p-Akt (p-Thr308), Bax, HIF1-α, p70S6 kinase beta (p-Ser423), and mTOR (p-Ser2448) were purchased from Signalway Antibody (Pearland, TX, USA). The antibodies against p53, Bcl2, caspase-3, and β-actin were purchased from Proteintech (Chicago, IL, USA). Antibody against Beclin 1 was purchased from Abcam (Cambridge, UK). Antibody against LC3 was purchased from Cell Signaling Technology (Beverly, MA, USA).

### Immunofluorescence assay

Skin fibroblasts were seeded in 6-well plates at a density of 5x10^5^ cells/well, incubated overnight, and then subjected to serum starvation or H_2_O_2_ treatment (900 μM) for 12 h or 24 h. Fibroblasts were then fixed in ice-cold 100% ethanol overnight, washed with phosphate buffered saline-Tween-20 (PBST), blocked with 5% milk in PBST, and incubated with anti-LC3 antibody (1:500) overnight. After washing, cells were incubated with Alexa 488 goat anti-mouse IgG (1:200). The fluorescence signal was measured and analyzed using a fluorescence microscope (Olympus IX71).

### Transmission electron microscopic examination

Fibroblasts were cultured in 60 mm dishes and subjected to starvation and H_2_O_2_ treatment (900 μM) for 12 h. Following treatment, cells were fixed in 4% paraformaldehyde in 0.1 M phosphate buffer for 4 h, washed with 0.1 M phosphate buffer, and then post-fixed in 1% OsO4 for 2 h. Specimens were then dehydrated with ethanol, embedded in Epon-812 resin, and polymerized for 2 days at 65ºC. Ultrathin sections (70 to 90 nm) were stained with uranyl acetate and lead citrate. The ultrathin sections were observed under a Hitachi-7000 electron microscope.

### Cell apoptosis assay

An Annexin V-FITC assay was used to quantify numbers of apoptotic cells by flow cytometry according to the manufacturer's instructions (Nanjing Keygen Biotech, KGA108). Briefly, cells were collected following trypsinization, washed twice with ice-cold PBS, and resuspended in 300 μL of 1× binding buffer containing 5 μL Annexin V-FITC and 5 μL PI for 30 min at room temperature in the dark. All samples were analyzed on a FACSCalibur flow cytometer (BD Biosciences) The results are expressed as early apoptotic cell percentages (PI-negative and Annexin V-positive cells). Apoptosis was detected based on nuclear morphology observed in cells stained with diaminopimelic acid (DAPI); cells with condensed and fragmented nuclei were considered apoptotic.

### Protein extraction and Western blot analysis

At the end of treatment, fibroblasts were lysed in lysis buffer (50 mM Tris–HCl pH 7.4, 150 mM NaCl, 1 mM EDTA, 1 mM EGTA, 1 μg/mL protease inhibitor cocktail, 5 mM phenylmethylsulfonyl fluoride, and 1 mM dithiothreitol containing 1% Triton X–100). Lysates were centrifuged at 10,000 × g for 10 min at 4°C, and protein concentration was determined. Samples (50 μg/lane) were resolved by 10% SDS-polyacrylamide gel electrophoresis and electrotransferred onto a polyvinylidene fluoride (PVDF) membrane. Blots were blocked for 1 h at 37°C in 20 mM Tris-HCl, pH 7.4, 150 mM NaCl, 0.02% Tween-20 (TBST) containing 5% skimmed milk and probed using a 1:1000 dilution of the appropriate primary antibodies (anti-LC3, Bax, Bcl-2, p70S6K, Akt, Akt^Ser473^ and Akt^Thr308^, β-Actin) and overnight incubation at 4°C. The blots were washed thrice with TBST and re-probed with goat-anti-mouse IgG or goat-anti-rabbit IgG conjugated to horseradish peroxidase (1:2000, Wuhan Boster Biotech, Wuhan, China).

Quantitative analyses of protein band optical intensities were conducted using the Kodak Gel Logic 4000 R Imaging System (Carestream, USA) and normalized to actin for protein expression or to total protein for protein phosphorylation.

### Statistical analysis

All data and results for skin fibroblasts were obtained from three independent primary cultures derived from three individual animals. The statistical significance of differences between means was assessed using one-way analyses of variance (ANOVA); *p* values <0.05 were considered statistically significant.
