# Correction: H_2_O_2_ treatment or serum deprivation induces autophagy and apoptosis in naked mole-rat skin fibroblasts by inhibiting the PI3K/Akt signaling pathway

**DOI:** 10.18632/oncotarget.18611

**Published:** 2017-06-27

**Authors:** Shanmin Zhao, Li Li, Shiyong Wang, Chenlin Yu, Bang Xiao, Lifang Lin, Wei Cong, Jishuai Cheng, Wenjing Yang, Wei Sun, Shufang Cui

**Present**: The images displayed in Figure 4.

**Correct**: The proper figure 4 appears below.

Original article: Oncotarget. 2016; 7:84839-84850. doi: 10.18632/oncotarget.13321

**Figure 4:**
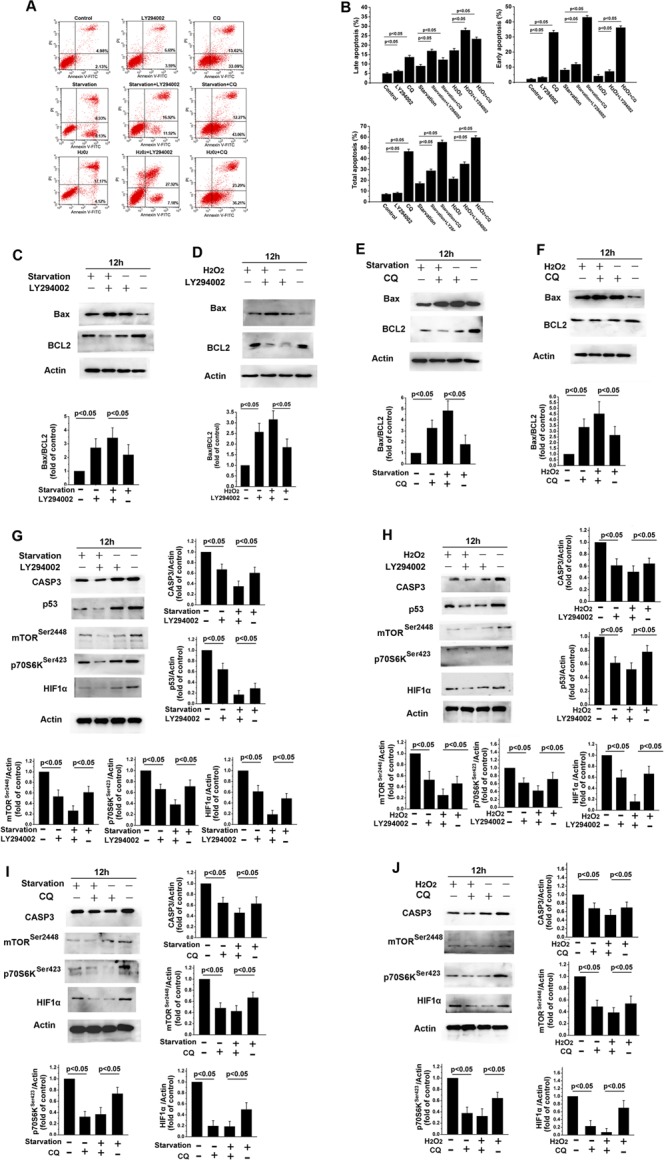
LY294002-induced inhibition of PI3K/Akt signaling increases apoptosis in skin fibroblasts (**A**) Flow cytometric analysis revealed that 12 h of treatment with LY294002 or CQ increased early and late apoptosis rates, and further increased starvation- or H_2_O_2_ treatment-induced increases in apoptosis rates, in skin fibroblasts. (**B**) Bar graph showing early and late apoptotic cell percentages. Means ± standard error are shown. (**C**–**J**) Western blots revealed that Bax levels increased, while Bcl2, p70S6K, p53, HIF1-α, and caspase-3 levels decreased, following 12 h of serum starvation or H_2_O_2_ treatment with or without LY294002 or CQ. Bar graphs show mean relative protein levels normalized to β-actin.

